# Synthesis and characterization of calcium and magnesium based oxides and titanates for photocatalytic degradation of rhodamine B: a comparative study

**DOI:** 10.1038/s41598-023-30013-3

**Published:** 2023-03-03

**Authors:** Naveensubramaniam Vijayakumar, Senthil Kumar Venkatraman, Syed Imthiaz, Elsayed A. Drweesh, Mohamed M. Elnagar, Sivasankar Koppala, Sasikumar Swamiappan

**Affiliations:** 1 Department of Chemistry, School of Advanced Sciences, Vellore Institute of Technology, Vellore, Tamil Nadu 632014 India; 2 Department of Inorganic Chemistry, National Research Centre, 33 El Bohouth St. (Former EltahrirSt.), Dokki, Giza, 12622 Egypt; 3 Faculty of Metallurgical and Energy Engineering, Kunming University of Science and Technology, Kunming, Yunnan 650093 China

**Keywords:** Chemistry, Materials science

## Abstract

The current investigation deals with the simple and ecological synthesis of CaO, MgO, CaTiO_3,_ and MgTiO_3_ for the photocatalytic dilapidation of rhodamine B dye. CaO was procured from chicken eggshell waste by calcination process, while MgO was produced by solution combustion method using urea as a fuel source. Furthermore, CaTiO_3_ and MgTiO_3_ were synthesized through an easy and simple solid-state method by mixing thoroughly the synthesized CaO or MgO with TiO_2_ before calcination at 900 °C. XRD and EDX investigations confirmed the phase formation of the materials. Moreover, FTIR spectra revealed the existence of Ca–Ti–O, Mg–Ti–O, and Ti–O which resembles the chemical composition of the proposed materials. SEM micrographs revealed that the surface of CaTiO_3_ is rougher with relatively dispersed particles compared to MgTiO_3_, reflecting a higher surface area of CaTiO_3_. Diffuse reflectance spectroscopy investigations indicated that the synthesized materials can act as photocatalysts under UV illumination. Accordingly, CaO and CaTiO_3_ effectively degraded rhodamine B dye within 120 min with a photodegradation activity of 63% and 72%, respectively. In contrast, the photocatalytic degradation activity of MgO and MgTiO_3_ was much lower, since only 21.39 and 29.44% of the dye were degraded, respectively after 120 min of irradiation. Furtheremore, the photocatalytic activity of the mixture from both Ca and Mg titanates was 64.63%. These findings might be valuable for designing potential and affordable photocatalysts for wastewater purification.

## Introduction

Despite being one of the most harmful pollutants, dyes are widely used in the fabric, food, plastic, chemical, and tabloid industries. Their discharge into the aquatic environment has a serious impact on living organisms^1,2^. Color reduces sunlight penetration through water, resulting in decreased photosynthetic activity and decreased biota development. In addition, dyes tend to bind metal ions, resulting in micro toxicity in fish and other living things^1,3^.

Normally, dyes are barely biodegradable and challenging to be eliminated by conventional approaches. In this context, rhodamine B (RhB) which belongs to the xanthene family, is a highly stable cationic dye due to its rigid heterocyclic structure^4^. Indeed, the high stability of RhB dye is beneficial for different industrial applications, however, makes its degradation not simple and challenging^5–7^. As a result, providing efficient, environmentally friendly, and cost-effective solutions for the breakdown of such contaminants is critical for the long-term viability of green habitats. This has resulted in a diverse range of techniques being employed to extract dyes from wastewater, including adsorption^8–10^, ultrafiltration^11^, chemical precipitation^12^, electrocatalytic breakdown^13^, and photodegradation^3,14–16^.

Photocatalytic degradation is potentially one of the cheapest, green, and most powerful techniques for the decontamination of water from dye pollutants. In other words, extremely oxidizing conditions can be established without any further required reagents, the only requirement is the supply of aerobic oxygen and a light irradiation source^17,18^. Electrons (e^−^), holes (h^+^), hydroxyl radicals (OH^·^), and superoxide radicals (O_2_^·−^) are all surface-active species that can be generated by photocatalytic degradation. The ability of the photocatalysts to generate surface-active species^19,20^. An assortment of materials with catalytic activity have been proposed for the photodegradation of pollutants such as graphitic carbon nitride (g-C_3_N_4_), TiO_2_, ZnO, CdS, CaO, MgO, CaTiO_3_, and MgTiO_3_, just to name a few^3,5,7,15,21–26^.

Compared to TiO_2_, the perovskite-type oxides based on titanium with a structural formula of ABO_3 _are attracting more and more attention in the last decade in photocatalysis due to their intriguing photophysical properties. CaTiO_3_ and MgTiO_3_ are utilized in a vast array of applications namely radar telecommunications, capacitors, thermistors, electronics, ceramics, superconductors, nonlinear optics, catalysis, piezoelectric, and dielectric devices^27–30^. Besides, they have high photocatalytic degradation activity towards different organic dyes^21,25^. Ca and Mg are among the most abundant metals on earth and their oxides can be also synthesized from waste materials. This makes them affordable for various important applications. Several methods such as hydrothermal, sol–gel, mechano-chemical, traditional solid-state, and polymeric precursor have been used for the synthesis of CaO, MgO, CaTiO_3_, or MgTiO_3_^7,31–34^. One should emphasize that the catalytic efficiency of these materials is highly reliant on the synthetic procedure and their precursors which of course have a direct impact on the final surface morphology, active sites, and physicochemical properties. Moreover, the performance of these photocatalysts extremely fluctuates from one dye to another^7,25,35^.

In this study, CaO derived from chicken eggshells waste as well as MgO were synthesized in their pure phases. Furthermore, CaTiO_3_ and MgTiO_3_ were obtained through a facile solid-state method by mixing thoroughly the synthesized CaO or MgO with TiO_2_ before calcination at 900 °C. Spectroscopic and microscopic techniques were used to comprehensively describe the synthesized materials. Moreover, we compared the photocatalytic activity of CaO, MgO, CaTiO_3_, and MgTiO_3_ on the degradation of rhodamine B (RhB) which is one of the most challenging dyes due to its high stability. The results might be beneficial for designing photocatalysts with tailored properties and high intrinsic efficiency.

## Results and discussion

### Characterization

#### Functional group evaluation

**Figure 1:**
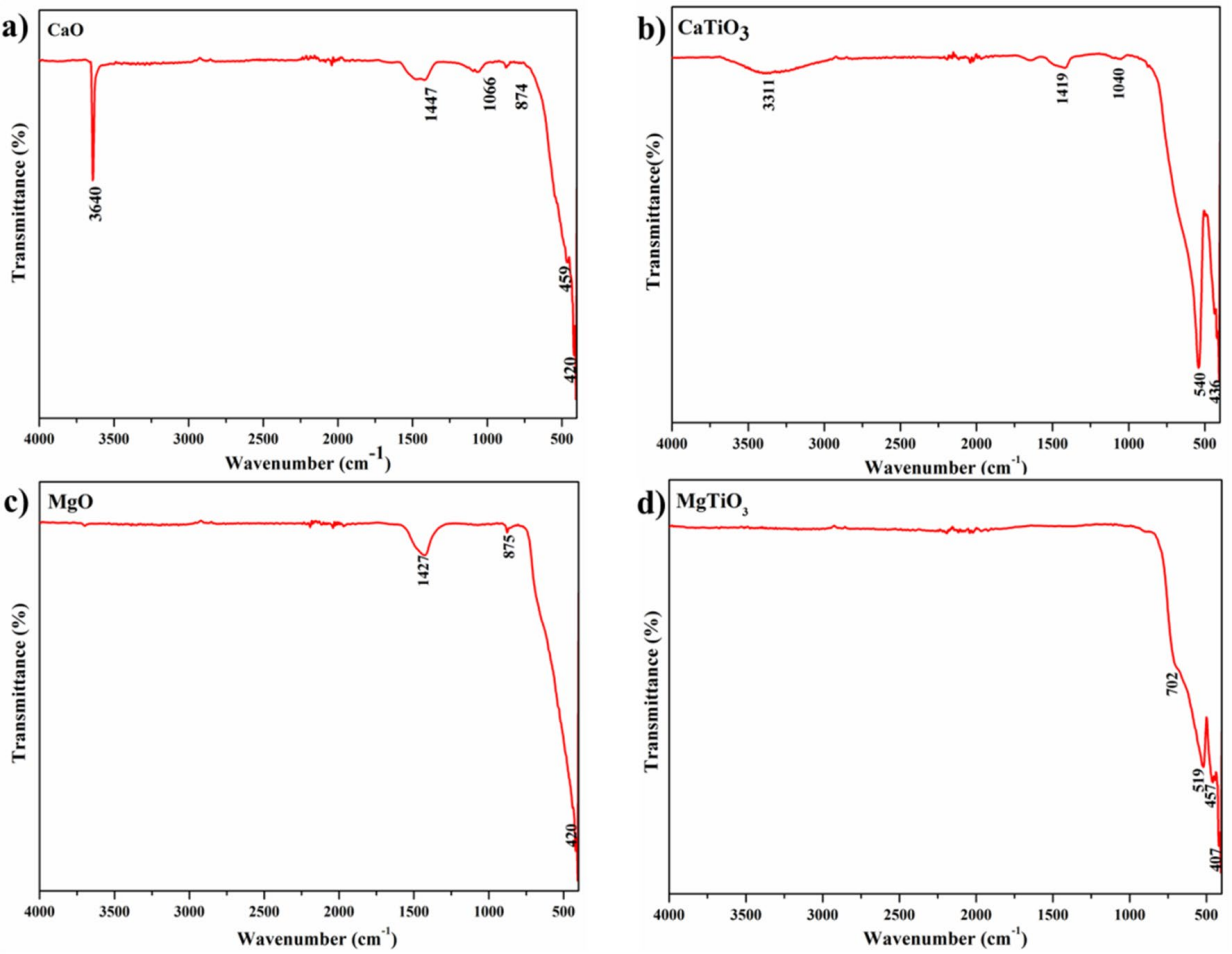
FT-IR spectra of (**a**) CaO, (**b**) CaTiO_3_, (**c**) MgO and (**d**) MgTiO_3_.

Figure. 1a–d shows the FT-IR spectra of CaO, CaTiO_3_, MgO, and MgTiO_3 _respectively. The FT-IR spectrum of CaO (Fig. 1a) displays a sharp peak at 3640 cm^−1^ that relates to the O–H stretching vibrations in Ca(OH)_2_. This highly intense peak is formed due to the presence of moisture in the crystal lattice caverns of the CaO system. The absorption peaks at 1477 and 874 cm^−1^ are attributed to the C–O asymmetrical and symmetrical vibrations. The bands that appeared at 459 cm^−1^ are due to the existence of Ca–O metal oxide which was transformed from CaCO_3_ in the eggshells^36,37^. In the case of CaTiO_3_, the broad peaks at around 3311 and 1419 cm^−1^ in Fig. 1b can be accredited to the hydroxyl group vibrations of the CaTiO_3_. Moreover, the distinctive bands at 540 and 436 cm^−1^ are typically known for CaTiO_3_ and they are corresponding to the Ca–Ti–O bending vibrations^35,38^.

The FT-IR spectrum of MgO in Fig. 1c shows a distinctive band at 420 cm^−1^ which is attributable to the presence of Mg–O metal oxide vibrations. Moreover, the absorption band at 1427 cm^−1^ is ascribed to the bending vibrations of the OH^−^^39^. However, in MgTiO_3_ bands that occurred at 519 and 457 cm^−1^ in Fig. 1d can be conferred to the Mg–Ti–O vibrations. Furthermore, the absorption bands at around 702 and 407 cm^−1^ are attributed to the Ti–O and Mg–O vibrations, accordingly^21,40^.

#### Powder XRD examination

**Figure 2:**
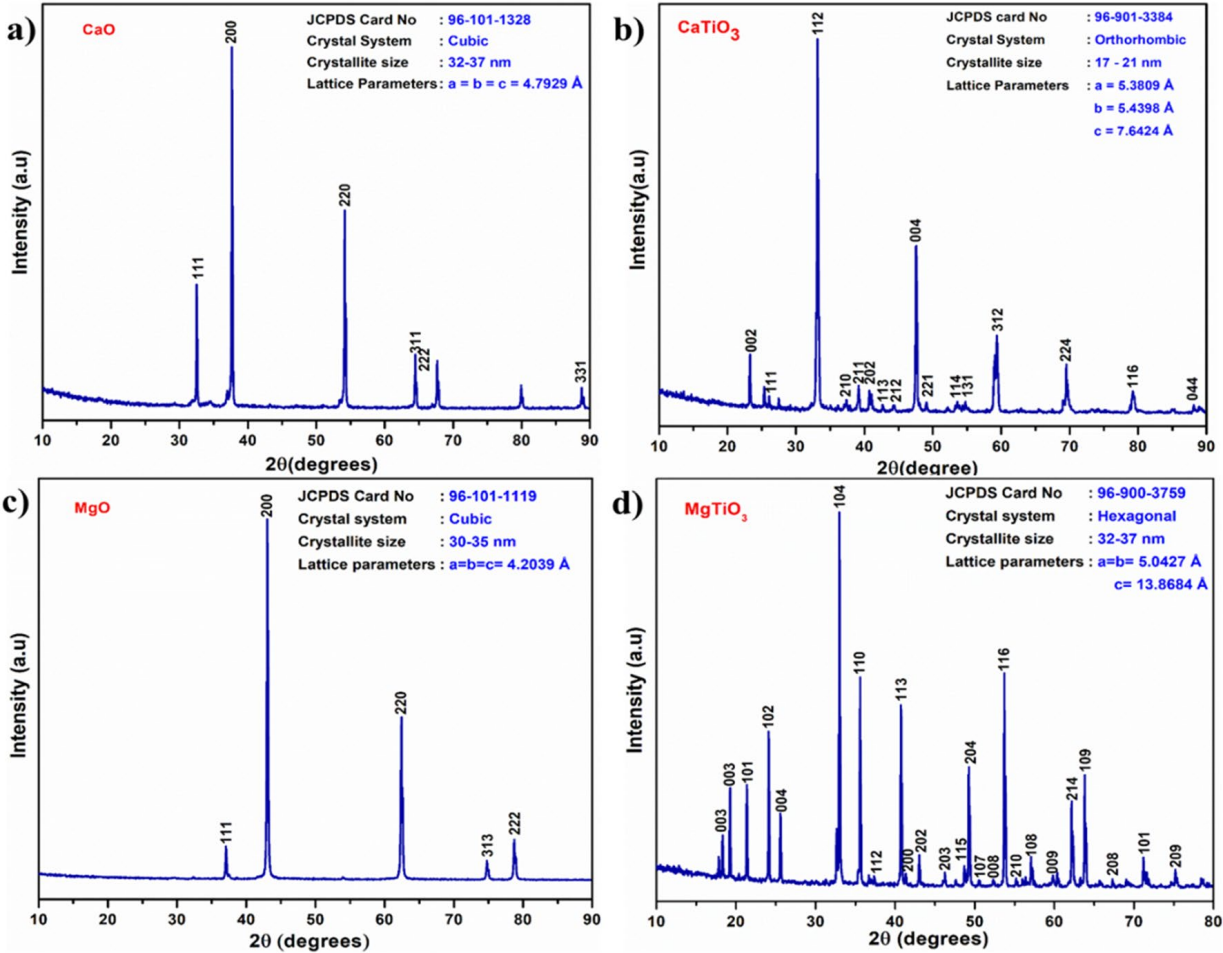
XRD patterns of (**a**) CaO, (**b**) CaTiO_3_, (**c**) MgO and (**d**) MgTiO_3_.

The XRD patterns of CaO, CaTiO_3_, MgO, and MgTiO_3_ are represented in Fig. 2. The XRD spectrum of CaO (Fig. 2a) exhibits intensive peaks at 2θ = 32.5°, 37.35°, and 53.85° that was matched and indexed to the JCPDS data card (96-101-1328) which fits in the cubic crystal structure. A mixture of CaO and Ca(OH)_2_ phases are observed; however CaO is the predominant material with lattice parameters of a = b = c = 4.7929 Å and crystallite size of 32–37 nm. For the XRD spectrum of CaTiO_3_ (Fig. 2b), the high-intensity peaks observed at 2θ = 33.18°, 47.58° and 59.39° match with the JCPDS card: 96-901-3384 proved the phase formation of pure CaTiO_3_.

The XRD pattern of MgO (Fig. 2c) illustrates the typical peaks of pure MgO that are similar to the JCPDS data card (96-101-1119). Furthermore, MgO has a cubic crystal structure with a crystallite size of about 30–35 nm and lattice parameter values of a = b = c = 4.2039 Å. Figure 2d shows the characteristic peaks of MgTiO_3_ at 2θ = 33.00°, 35.62°, 40.67°, and 53.77° that match with the JCPDS card: 96-900-3759 and indicate the synthesis of MgTiO_3_ with the hexagonal crystal arrangement. Table. 1 shows the computed lattice characteristics and crystalline size of the produced materials.

**Table 1:** Lattice parameters, crystallite size, and crystal structure of the synthesized materials.

Materials	Cell parameters (Å)			Crystallite size (nm)	Crystal structure
	A	B	c		
CaO	4.7929	4.7929	4.7929	32–37	Cubic
MgO	4.2039	4.2039	4.2039	30–35	Cubic
CaTiO_3_	5.3809	5.43982	7.6424	17–21	Orthorhombic
MgTiO_3_	5.0427	5.0427	13.8684	32–37	Hexagonal

The crystalline size was computed using the Debye-Scherer formula as follows:

$$\mathrm{D }= \frac{0.89\uplambda }{{\upbeta \cos\uptheta }}.$$

In addition, the lattice parameters were determined utilizing the given expression:

$${1}/{\text{d}}^{{2}} = {1}/{\text{sin}}^{{2}} \upbeta ({\text{h}}^{{2}} /{\text{a}}^{{2}} + {\text{k}}^{{2}} {\text{sin}}^{{2}} \upbeta /{\text{b}}^{{2}} + {\text{l}}^{{2}} /{\text{c}}^{{2}} - {\text{2hkcos}}\upbeta /{\text{ab}}),$$

$${\text{d = }}\chi /{\text{2 sin}}\theta ,$$

where D is the average crystallite size, β corresponds to the full width at half maximum, and θ indicates Bragg’s angle.

#### SEM/EDX analysis

**Figure 3:**
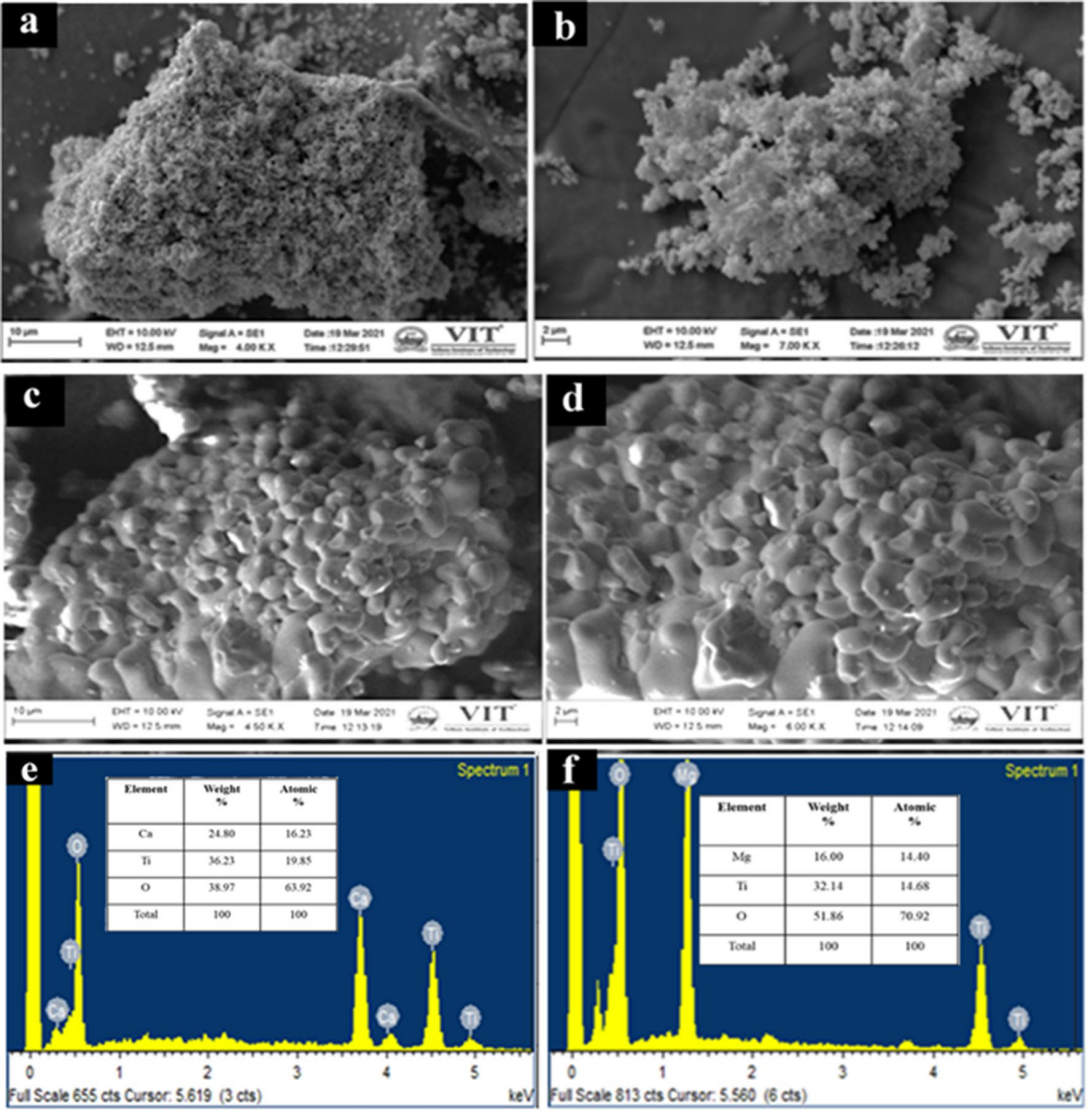
SEM micrographs of (**a,b**) CaTiO_3_ and (**c,d**) MgTiO_3_ and their corresponding EDX spectra are shown in (**e,f**), respectively.

 The morphology and elemental composition of CaTiO_3_ and MgTiO_3_ were investigated by SEM and EDX. As illustrated in Fig. 3a,b, CaTiO_3_ micrographs reveal clumps of particles with a rough surface and an uneven shape. In comparison, MgTiO_3_exhibits a smoother surface with a spherical beads like structure over the surface of the material with non-homogeneous pores distributed throughout the surface (Fig. 3c,d). The SEM images demonstrate the porous nature of the particle which might be due to the elimination of impurities during calcination. The micrographs illustrate that the surface morphology of CaTiO_3_ and MgTiO_3_ are distinct, although they were formulated under identical preparatory circumstances. These observations indicate that alkaline earth metals have a significant impact on the final surface morphology of titanates.

The EDX spectra of CaTiO_3_ and MgTiO_3_were exemplified in Fig. 3e,f, respectively. The peaks observed from the EDX spectrum divulge the existence of the peaks related to Ca, Mg, Ti, and O elements in a stoichiometric ratio. No other elemental peaks were found in the spectrum which indicates the formation of respective titanates without any impurities.

#### UV-DRS reflectance spectroscopy studies

**Figure 4:**
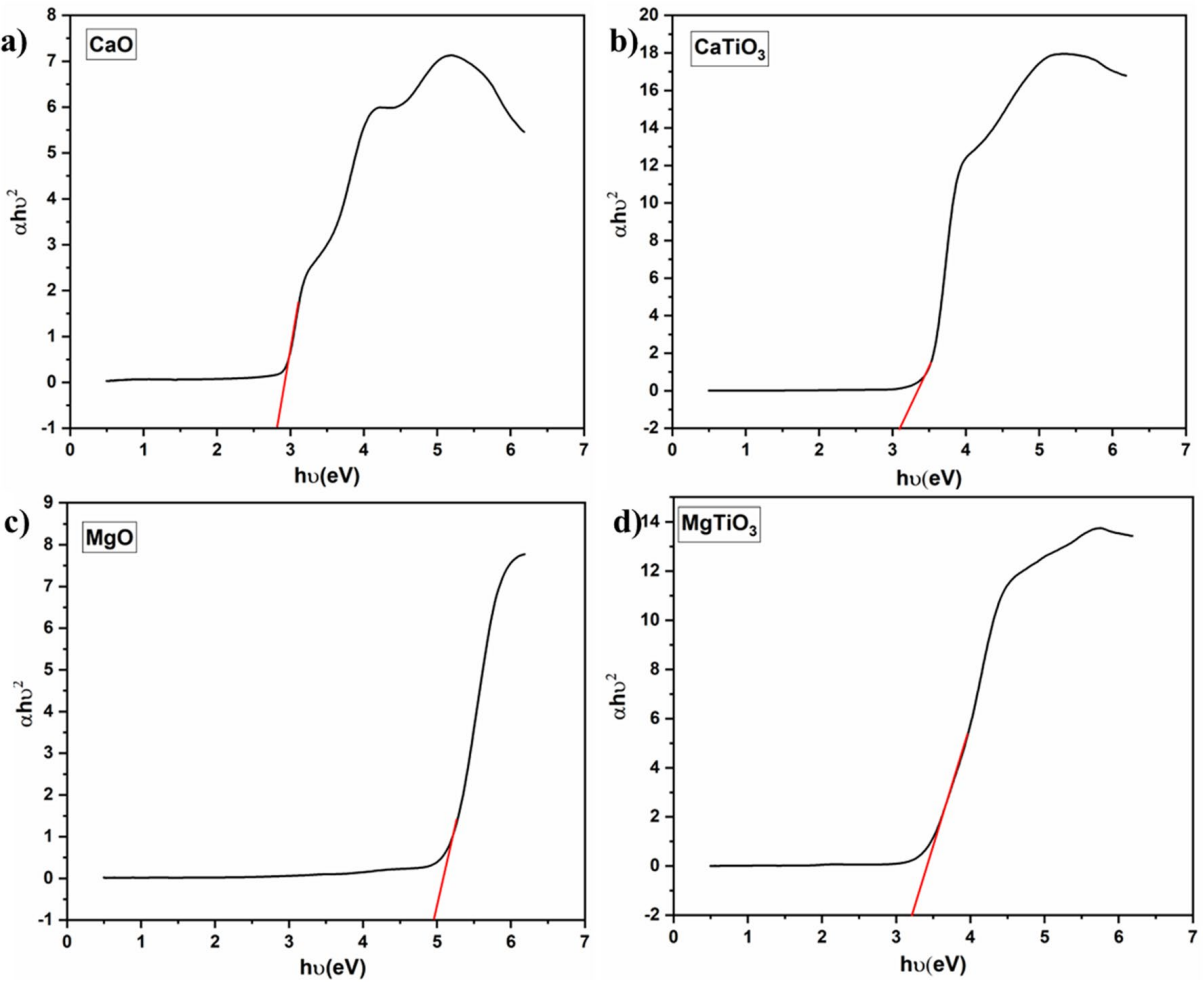
Tauc’s Plots for bandgap estimation of (**a**) CaO, (**b**) CaTiO_3_, (**c**) MgO and (**d**) MgTiO_3_.

To identify which fraction of the sun spectrum may be absorbed by semiconductors, it is necessary to calculate the optical bandgap energy (E_g_). The diffuse reflectance experiments were used to estimate the *E*_*g*_ of CaO, MgO, CaTiO_3_, and MgTiO_3_ from the absorption spectrum (Fig. 4a–d). Furthermore, the E_g_ was calculated using the transition rate expression for direct bandgap semiconductors. The absorption coefficient for direct bandgap measurements was given by Tauc’s equation:

$$\left( {\alpha h\nu } \right)^{n} = \, A \, \left( {h\nu \, {-} \, E_{g} } \right),$$

where α is the absorption coefficient, *hν* is the photon energy*,* and A is a constant. The exponent *n* in Tauc’s equation depends on the types of transitions^41,42^. Figure 4a,b validate that, the *E*_*g*_value for CaO calculated from Tauc’s plot is 2.80 eV versus SHE, whereas in case of MgO it was found to be around 5 eV. On the other hand, the *E*_*g*_values for CaTiO_3_ and MgTiO_3_ were observed to be 3.0 eV and 3.12 eV, respectively which are slightly higher than those of their oxides. The *E*_*g*_value for CaTiO_3_ was reported to be in the range of 3.0–3.6 eV versus SHE, while the *E*_*g*_value for MgTiO_3_ was in the range of 3.0–3.2 eV versus SHE^21,36,39,43^.

### Photocatalytic activity

**Figure 5:**
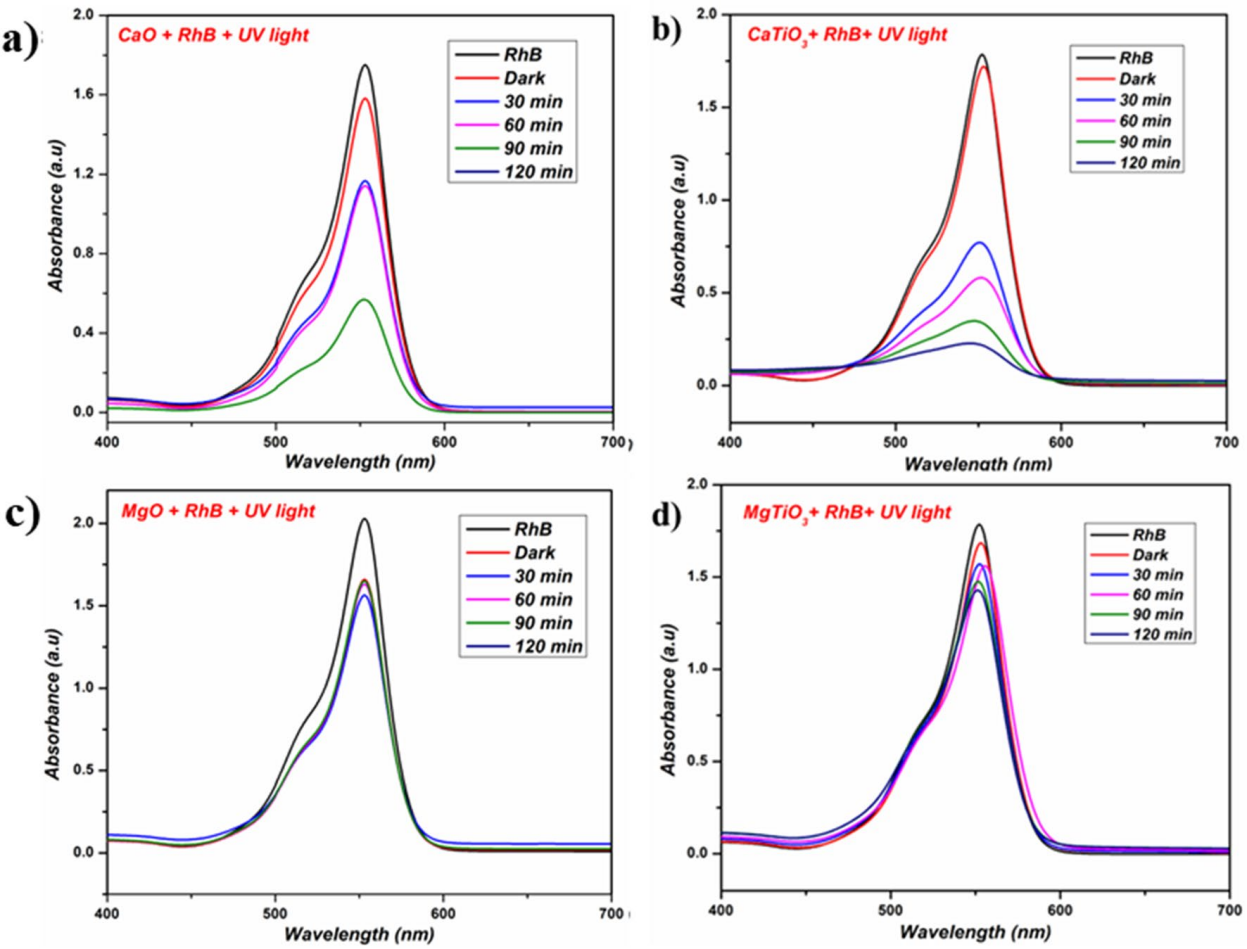
UV–Visible spectra for the disintegration of RhB dye by (**a**) CaO, (**b**) CaTiO_3_, (**c**) MgO, and (**d**) MgTiO_3_.

**Figure 6:**
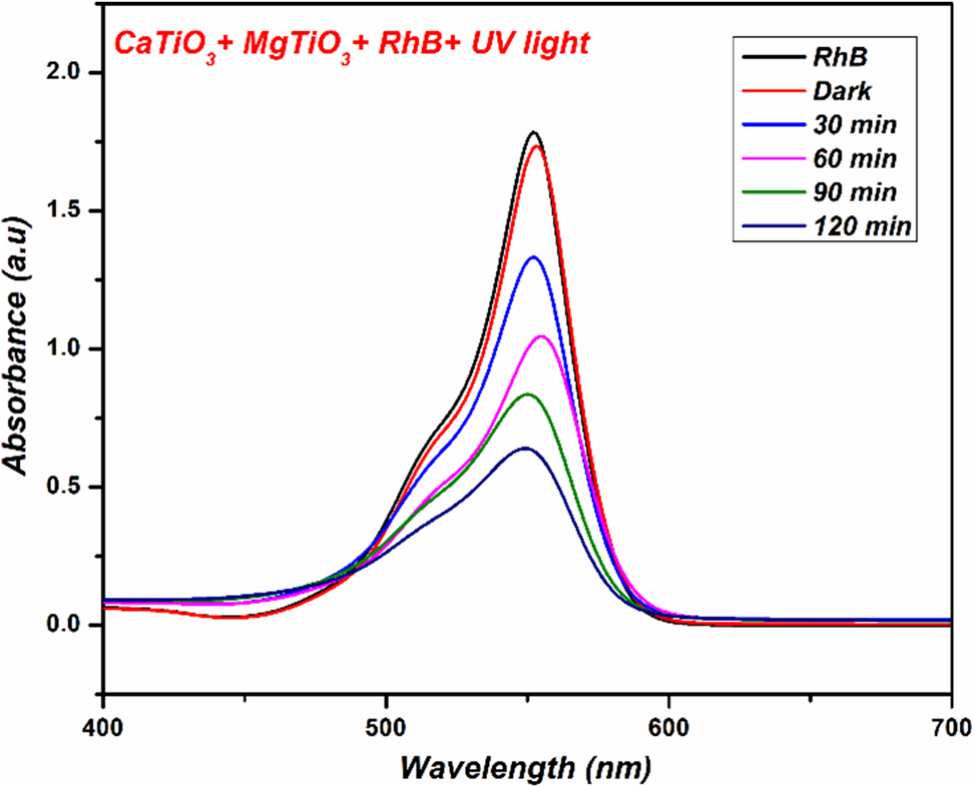
UV–Visible spectra for the disintegration of RhB dye by Mixture (CaTiO_3_ + MgTiO_3_).

Employing ultraviolet illumination, the photocatalytic activity of the newly synthesized CaO, MgO, CaTiO_3,_ MgTiO_3_ and the mixture of respective titanates was investigated for the degradation of RhB dye in water. As a function of the irradiation period (30–120 min), the dilapidation ability of the as-synthesized materials against RhB dye was compared using the UV–Vis spectra exhibited in Figs. 5a–d and 6. In addition, the absorption of rhodamine B dye has been associated with absorption peaks at 503 and 550 nm, which have been ascribed to the absorption of the de-ethylated and tetra ethylated rhodamine B dye, respectively^44^. Furthermore, the RhB concentration is proportional to its absorbance; thus, the changes in the absorption peak value at λ_max_ = 550 nm were monitored during the photocatalytic degradation reaction to evaluate the performance of the proposed photocatalysts. The photodegradation efficacy (% E) was calculated using the following equation^45^:

$$\% {\text{ E }} = \, \left( {{1} - {\text{A}}_{{\text{t}}} /{\text{A}}_{{\text{o}}} } \right) \, \times { 1}00,$$

where A_t_ is the absorbance after *t* min and A_0_ is the absorbance at time *t* = 0.

**Figure 7:**
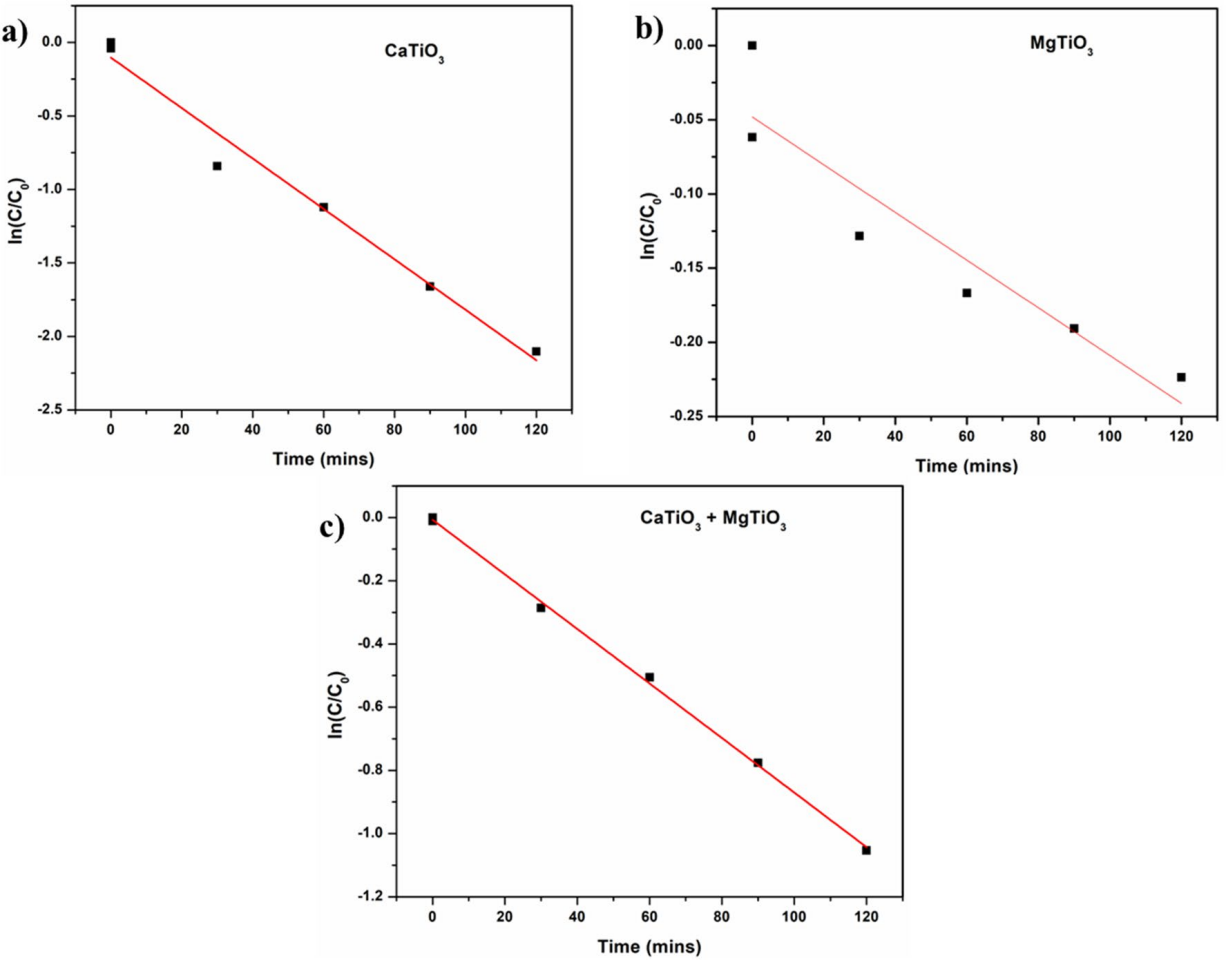
Kinetic plot of (**a**) CaTiO_3_, (**b**) MgTiO_3_ and (**c**) CaTiO_3_ + MgTiO_3_.

As a control experiment, the adsorption of RhB on the synthesized materials was tested in the darkness for 120 min. According to the results, the intense peak at λ_max_ = 550 did not change in indicating that the catalysts do not adsorb RhB. Instead, the intensity of the dye absorption peaks under UV light irradiation intensively decreases in the case of CaO and CaTiO_3_ even after illumination for 30 min which is correlated to their photocatalytic activity. Furthermore, the photocatalytic activity of CaO and CaTiO_3_ increases overtime to degrade about 63.13 and 87.64% of RhB solution at 120 min, respectively. In comparison, the synthesized MgO and MgTiO_3_ do not have high efficacy to deteriorate the dye. The rate of RhB degradation is slow since only the photocatalytic activity of MgO and MgTiO_3_ are 21.39 and 29.44%, respectively. Whereas the mixture of both the titanates showed the degradation of about 64.62% on UV-light irradiation. The comparative degradation of RhB dye is demonstrated in Table 2 which is given below. From the above spectral data, we plot ln (C/C_o_) vs time for a detailed comparison of the proposed catalyst under UV radiation in Fig. 7 and the plots follow first-order rate kinetics.

**Table 2:** Rhodamine B dye decomposition (%).

Dye	Light source	Volume (mL)	Catalyst	Degradation (%)
Rh B	UV-Light	200	CaO	63.13
Rh B	UV-Light	200	CaTiO_3_	87.64
Rh B	UV-Light	200	MgO	21.39
Rh B	UV-Light	200	MgTiO_3_	29.44
Rh B	UV-Light	200	CaTiO_3_ + MgTiO_3_	64.62

Compared to Mg–O, Ca–O has a reduced bond strength^46,47^ and more active sites are formed during bond cleavage, which is a more crucial aspect in photocatalytic activity^16^. Furthermore, it is obvious from SEM micrographs (Fig. 3) that the surface of CaTiO_3_ is rougher than that of MgTiO_3_indicating that CaTiO_3_ has a greater surface area. The increased surface area provides further accessible active sites for the photoreduction efficiency of RhB. Moreover, the deterioration efficiency of titanites is higher than that of their corresponding metal oxides, indicating the synergistic effect of Ti in improving the photoactivity of CaO and MgO. One should emphasize that the photocatalytic activity of CaO, MgO, and their titanites strongly depends on several variables such as their synthetic procedure and the investigated dyes. For example, CaO NP derived from eggshell showed high activity towards the photodegradation of methylene blue and toluidine blue compared to a neglectable activity towards RhB^7^. Interestingly the CaO synthesized in this study through a facile and affordable solid-state method shows a high activity towards the photodegradation of RhB. MgO showed a high photocatalytic degradation activity against reactive yellow (RY) dye^43^. Furthermore, MgO nanoflakes exhibited excellent performance for the dye degradation of methylene blue and methylene orange^48^. Moreover, several reports were available on the degradation of RhB employing pure and mixture materials^49–51^. In comparison, it seems that the combustion synthesis of MgO resulted in agglomerated particles with poor photocatalytic activity towards the photodegradation of RhB in this study. However, it might be beneficial to investigate the photocatalytic activity of the synthesized MgO and MgTiO_3_ towards different dyes. These fundamental insights might help to design photocatalysts with tailored properties and high intrinsic activity.

### Chemical oxygen demand (COD)

**Figure 8:**
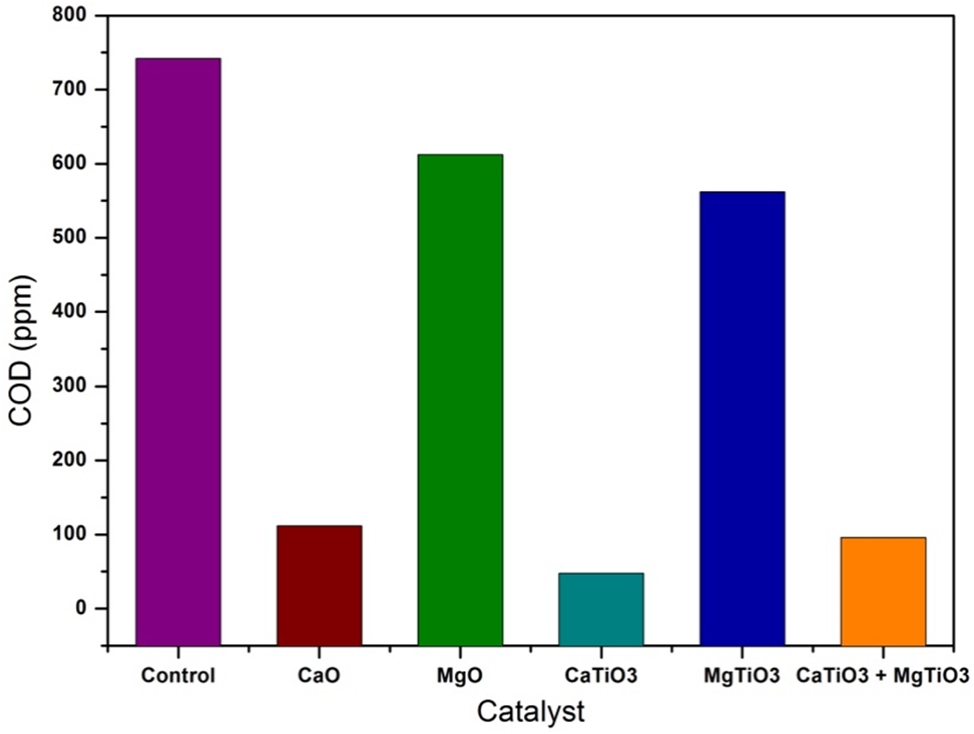
COD of degraded RhB dye with calcium/magnesium oxides and titanates catalyst under UV radiation.

A COD experiment was conducted to verify that the decolonization of RhB is induced by photocatalytic degradation and not by adsorption. After 2 h treatment, the COD values of the samples treated with CaO, MgO, CaTiO_3_, MgTiO_3_, and CaTiO_3_ + MgTiO_3_ were determined to be 112, 612, 48, 562, and 96 mg L^−1^, respectively, as illustrated in Fig. 8. These results showed a significant decrease in the COD value obtained for the sample treated the catalysts compared to the untreated one (742 mg L^−1^). The observation mentioned above demonstrated using less K_2_Cr_2_O_7_ to oxidize the organic dye pollutants found in the samples. The decrease in COD values proved that the RhB dye was photocatalytically degraded when exposed to UV light. The decline in COD value revealed a trend that was consistent with UV analysis. The catalytic effectiveness of the aforementioned catalysts was demonstrated by the consumption of K_2_Cr_2_O_7_ for the oxidation of treated samples.

## Experimental section

### Materials and methods

All chemicals including Mg(NO_3_)_2_·6H_2_O(98%), TiO_2_ (99%), urea (99%), Nitric acid (HNO_3_) (68%), were of analytical gradient and employed as obtained from SDFCL (SD Fine Chemical Limited) without further refinement.

### Synthesis of metal oxides and their corresponding titanates

#### Preparation of eggshell derived calcium oxide

CaO was extracted from chicken eggshell waste according to the reported procedures in the literature^52,53^ with some modifications as follows: To eliminate suspended impurities from any organic phases and pathogens, the eggshells were first cleansed with double-distilled water before being seethed at 100 °C for 60 min. The washed eggshells were dehydrated in a hot air oven at 150 °C for 24 h and subsequently were ground manually into a fine powder in a pestle and mortar. After that, the eggshells were calcined in air at 900 °C for 2.5 h to obtain eggshell derived calcium oxide and grounded manually into a fine powder.

#### Preparation of magnesium oxide

MgO was created utilizing urea as fuel and nitric acid as an oxidizing agent via the solution combustion method. 7.69 g of Mg(NO_3_)_2_·6H_2_O were thoroughly mixed with 3.00 g of urea by stirring in 30 mL of double-distilled water and then 5–6 drops of concentrated nitric acid were added. Afterward, the solution (without drying) was moved into a beaker and heated in a pre-heated furnace at 400 °C for 30 min. Finally, the obtained powder was calcined at 900 °C for 6 h to achieve the pure phase of MgO then, it was cooled down to room temperature and finally ground into a fine powder.

#### Synthesis of calcium and magnesium titanate

Both CaTiO_3_ and MgTiO_3_were synthesized from their corresponding metal oxides through the traditional solid-state approach. TiO_2_ was used as starting reactant material along with eggshell-derived CaO and MgO, respectively. The synthesized CaO (2.80 g) was mixed and ground thoroughly with TiO_2_ (3.99 g) before sintering at 900 °C for 6 h. The latter, MgTiO_3_ was synthesized by grinding the synthesized MgO (2.01 g) with TiO_2_ (3.99 g). The homogenous powder was then calcined at 900 °C for 6 h to acquire the pure materials of the corresponding titanates; CaTiO_3_ and MgTiO_3_.

### Characterization

A Shimadzu IR Affinity-1S CE FT-IR spectrophotometer in the range of 4000–500 cm^−1^ was employed for the functional group analysis of the respective titanates and oxides. The presence of the pure phases of CaO, MgO, CaTiO_3_, and MgTiO_3_ was confirmed by the XRD analysis. The XRD patterns were obtained by a D8 advance powder X-ray diffractometer (Bruker AXS GmbH, Karlsruhe, Germany) with Cu/Kα radiation filter at a wavelength of 1.5406 Å. An EVO 18 Research (Zeiss India) SEM equipped with an energy dispersive X‐ray analysis unit operating at a hastening potential of 20 kV was used for accomplishing the surface morphology along with the elemental composition of the synthesised materials. The photosensitive characteristics of the materials were determined with the aid of UV–Vis diffuse reflectance spectroscopy (Jasco, V-670).

### Photocatalytic experiments

To evaluate the degradation capability of the as-prepared materials, 50 mg of the catalysts were supplemented to 100 mL of the 10 ppm rhodamine B dye solution which was prepared from the stock solution. The dye solution and the catalyst were mixed using the magnetic stirrer in a dark condition to bring up the adsorption–desorption between them. Then the mixture was placed inside the Heber immersion type photoreactor in a dark condition and exposed to the UV light irradiation of wavelength 300–400 nm. Then, 10 mL of the solution was stockpiled every 30 min (30, 60, 90, and 120 min) to test the degradation activity as a function of time and the Chemical oxygen demand (COD) was conducted as per the procedure reported earlier^16^.

## Conclusions

The current work dealt with the preparation of calcium and magnesium titanates from their respective oxides using the solid-state approach for the photoreduction of RhB dye under UV–Visible irradiation. The phase formation and purity of the materials were confirmed by the FTIR, XRD, and EDX analyses. SEM micrographs reveal that the surface of MgTiO_3_ is smoother with agglomerated particles compared to CaTiO_3_. Diffuse reflectance spectroscopy studies indicate the higher bandgap energy of titanites compared to their alkaline metal oxides. However, the catalytic efficacy of titanates is higher compared to their corresponding alkaline metal oxides, indicating the synergistic effect of Ti. Furthermore, the photocatalytic degradation performance of Ca-based oxides and titanites is much higher than that of Mg-based ones. This might be due to the higher bond strength of Mg–O than Ca–O which in turn makes the bond cleavage of Mg–O much more difficult. Additionally, the surface morphology of CaTiO_3_ and MgTiO_3_ should be considered whereas, the agglomeration is higher in the case of MgTiO_3_ which, indeed decreases the active sites. However, the activity of the MgO might be improved by optimizing the synthetic procedure such as the choice of fuel and reduction in the materialization temperature. Since the photodegradation activity of the materials changes from one dye to another, it is worth trying the photocatalytic activity of the proposed material towards less stable dyes. All of these aspects are valuable for designing effective photocatalysts with tailored properties.

## Data Availability

Data will be made available on reasonable request from the corresponding author.
